# DDT Paradox: Bouwman et al. Respond

**DOI:** 10.1289/ehp.1103957R

**Published:** 2011-10-01

**Authors:** Henk Bouwman, Henk van den Berg, Henrik Kylin

**Affiliations:** North-West University, Potchefstroom, South Africa, E-mail: henk.bouwman@nwu.ac.za; Wageningen University, Wageningen, the Netherlands; Linköping University, Linköping, Sweden

In our commentary (Bouwman et al. 2011), we presented our centrist point of view on DDT, briefly, that despite DDT’s known protective effects against malaria, there is a need to eventually eliminate its use due, in part, to growing concerns about DDT’s human health impacts. How this can be misrepresented as anti-DDT by Tren and Roberts is simply astounding.

The reference to “isolated studies” on health aspects of DDT by Tren and Roberts has no basis. Of the 22 epidemiological studies from 2009 that we cited, 12 showed that DDT was significantly associated with some condition. We also notice that their “thousands of studies” is not substantiated by references. The evidence we presented is consistent with that of Eskenazi et al. (2009) and justifies our recommendation to invoke precaution.

Tren and Roberts refer to the recent Convention of the Parties of the Stockholm Convention (COP-SC) and the DDT Expert Group’s report to the COP-SC (UNEP 2011b). The report stated that

In certain settings, there is a continued need for DDT for malaria vector control, until locally appropriate and cost-effective alternatives are deployed for a sustainable transition away from DDT. (UNEP 2011b)

Moreover, the COP-SC report (UNEP 2011a) stated that “there was broad support for the recommendation by the DDT expert group that DDT was needed in some countries for disease vector control.” It is simply impossible to construe this statement as “anti-DDT.”

Most, if not all, of the actions considered by Tren and Roberts as “anti-DDT” can be aligned with a centrist point of view, because most countries involved are Parties to the SC. The COP-SC final report (UNEP 2011a) stated that “there was broad agreement regarding the need to combat malaria and to reduce and eventually eliminate the production and use of DDT.”

Regarding the World Health Organization (WHO) assessment of DDT (WHO 2011) quoted in their letter, Tren and Roberts fail to add the qualification included in the same paragraph, namely,

In some areas, the exposures in treated residences have been higher than potential levels of concern. Efforts are needed to implement best practices to protect residents in treated households from exposures arising from IRS [indoor residual spray]. Of particular concern would be women of childbearing age who live in DDT IRS-treated dwellings and transfer of DDT and DDE to the fetus in pregnancy and to the infant via lactation.

This is what we concluded in our commentary (Bouwman et al. 2011).

WHO procedures recommend the removal of furniture and food from houses to be sprayed, as well as a no-entry period (Najera and Zaim 2002). This implies an explanatory obligation toward the households why this has to be done. Nowhere in our commentary did we actually argue “that households should be informed about” the possible effects of DDT, as purported by Tren and Roberts. We maintain however, that the use of any insecticide in IRS raises ethical issues. This requires further investigation; the implications for IRS are yet unknown.

We defined our position as centrist because we acknowledge the role of DDT in malaria vector control as well as the urgency to move away from DDT once suitable, safe, and sustainable alternatives are in place. Our position is based on available evidence; invoking precaution, we suggest, is the best approach to address the paradox.
